# Microarray-based screening system identifies temperature-controlled activity of Connexin 26 that is distorted by mutations

**DOI:** 10.1038/s41598-019-49423-3

**Published:** 2019-09-19

**Authors:** Hongling Wang, Frank Stahl, Thomas Scheper, Melanie Steffens, Athanasia Warnecke, Carsten Zeilinger

**Affiliations:** 1 Hannover Medical School, Department of Otorhinolaryngology, Head- and Neck-Surgery, Carl-Neuberg-Str. 1, 30625 Hannover, Germany; 2 Gottfried-Wilhelm-Leibniz University of Hannover, Institut für Technische Chemie/BMWZ (Zentrum für Biomolekulare Wirkstoffe), Callinstr. 5, 30167 Hannover, Germany; 3 Gottfried-Wilhelm-Leibniz University of Hannover, BMWZ (Zentrum für Biomolekulare Wirkstoffe), Schneiderberg 38, 30167 Hannover, Germany; 4 Cluster of Excellence EXC1077 “Hearing4all”, German Research Foundation (DFG; “Deutsche Forschungsgemeinschaft”), Hannover, Germany

**Keywords:** Porins, Molecular medicine

## Abstract

Here, we show that human Connexin 26 (hCx26 or Cx26WT) hemichannel opening rapidly enables the transport of small molecules when triggered by temperature and by compensation of the Ca^2+^ blockade with EDTA. Point mutations within Cx26 were analysed by a novel optical microarray-based Lucifer Yellow uptake assay or by two electrode voltage clamp (TEVC) on frog oocytes to monitor simultaneous activities of channel proteins. Point mutations L90P, F161S, R184P or K188N influenced the temperature-dependent activity drastically. Since several mutations blocked trafficking, the temperature-dependent activity of the recombinant synthesized and purified wild-type Cx26WT and Cx26K188N hemichannel was tested by liposome flux assay (LFA) and on a microarray-based Lucifer Yellow uptake assay under warm conditions (>30 °C). The data from TEVC measurements and dye flux experiments showed that the mutations gave no or only a weak activity at increased temperature (>30 °C). We conclude that the position K188 in the Cx26WT forms a temperature-sensitive salt bridge with E47 whereas the exchange to K188N destabilizes the network loop- gating filter, which was recently identified as a part of the flexible Ca^2+^ binding site. We assume that the temperature sensitivity of Cx26 is required to protect cells from uncontrolled release or uptake activities through Cx26 hemichannels.

## Introduction

Hearing loss is the most common neurodegenerative disorder affecting nearly 7% of the world population^1^. This results in more than 460 million hearing impaired people world wide and produces - if left untreated- an economical annual burden of more than US$ 750 billion. Many causes for hearing loss have been described, e.g. genetic predisposition, ototoxic substances, immunologic or environmental factors as well as aging. Of the genetic causes, mutations of connexin are most common^2^.

Connexins are proteins involved in wound repair, cardiac disease or in tumour suppression. Healthy development and homeostasis are based on proper function of connexins^3–5^. Defects or mutations are linked to several diseases referred to as connexinopathies and accompanied by different molecular changes mediated by inactivity of the channel, unregulated activity of the channel, lack of gap junction formation or lack of regular trafficking to the plasma membrane and formation of aggregates^6^. Mutations can affect different genes coding for connexins such as GJB2, GJB3 and GJB6^7,8^. The respective connexins (Cx26, Cx30 and Cx31) are expressed by cochlear tissue. The mode of inheritance is mainly autosomal recessive. The hearing loss that results from connexon mutations can vary^9,10^. Not only congenital forms exist but also late onset progressive hearing loss^11–16^. Contrary to initial assumptions of hair cell loss due to connexon mutations, cochlear cellular architecture is still preserved in Cx26 mutations with late onset hearing loss^12^. Disturbance of the endocochlear potential is the leading pathomechanism for hearing loss in these patients^17–20^.

Connexins are the only protein family that is known to form gap junctions for direct intercellular communication^21^. In humans they constituted by of 21 different proteins. All connexins share a conserved structure of two extracellular loops, four alpha-helical transmembrane regions, one cytoplasmic loop, and a cytoplasmic N- and C-terminal domains tail^22,23^. Six connexins assemble into hexameric pores known as connexin hemichannels or connexons (homomeric in the case of identical connexins and heteromeric in the case of different connexins). Two hemichannels connect to each other forming dodecameric cell-cell connections, i.e., gap junctions, either homotypic (consisting of identical hemichannels) or heterotypic (consisting of different hemichannels)^4,23^. Their composition enables an intermixing that results in an immense spectrum of channel diversity and selectivity coordinates the transport of many thousands of small molecules from one cell to neighbouring cells^24^. The nomenclature of the connexins is based on their predicted size, which is determined by the lengths of the cytoplasmatic loop and the cytoplasmatic C-terminal tail, e.g., Cx43 is a 43 kDa protein. Based on homology, each connexin belongs to one of the five (alpha to epsilon) subclasses. The nomenclature of the gene is based on the gap junction (GJ) followed by the letter that indicates their subclass (alpha to epsilon), and a number that indicates the order of discovery. For example, GJB2 indicates the gene name of Cx26.

In depth knowledge of the function of connexons and gap junctions may present an avenue towards novel therapeutic approaches for restoring hearing in patients with GJB2 mutations. Several effects of mutations in Cx26 with clinical relevance were identified by dye transport localization tests or electrophysiological studies. Deafness mutations such methionine position 34 to threonine (M34T), leucine position 90 to proline, arginine position 217 to histidine, phenylalanine position 161 to serine and arginine position 184 to proline (L90P, R127H, F161S, and R184P) affect the function of gap junction channels at different levels of protein expression; other positions have been identified as relevant for non-syndromic, sensorineural hearing loss^25,26^. Residues in the highly conserved TM4 domain may be relevant for trafficking since some of these amino acid positions range from being absolutely conserved lysine position 188 (K188), strictly conserved phenylalanine position 191 and leucine position 205 (F191, L205), moderately conserved methionine position 195, isoleucine position 203 and asparagine position 206 (M195, I203, N206), somewhat conserved alanine position 197, serine position 199, glycine position 200, cysteine position 202 and asparagine position 206 (A197, S199, G200, C202, N206) to one appearing solely in human Cx26 threonine position 208 (T208)^27^. Exchange of small molecules and ions enabled by gap junctions and underlies two different gating processes: slow-loop gating and fast voltage dependent gating and can be triggered differentially^28–30^. Hitherto it is unknown how the passage ions and molecules is differentially modulated perhaps according to their size^31,32^.

The open channel mediates transport of molecules of up to ~1.2 kDa in size^30,31^. Depolarizing voltage pulses are used as a regulator of the open probability to activate outwardly rectifying currents of hemichannels, whereas hyperpolarizing voltage pulses deactivate the hemichannels. The structural comparison of Cx46/50 obtained from cryo crystallization experiments with the Cx26 structure revealed that this filigree structure is disturbed when conserved amino acid positions required for subunit interaction, gap junction formation, electrical, chemical or thermo sensing mutated^32^.

In order to understand the effect of mutations for the development of possible therapeutic strategies, the Cx26 hemichannels were characterized and it was shown recently that the activity of human Cx26 hemichannels are responsive to temperature^33,34^. Furthermore, analysis by high-resolution Raman spectroscopy revealed conformational changes in the presence of phosphatidylcholine (POPC) lipid, Ca^2+^ ions, and by temperature, indicating that the temperature-dependent activity results from the protein activity and not from internal cellular activities or trafficking^34^. The reason for this relevant observation was unclear as a thermo sensor is not known. Therefore, we first calculated the Q_10_ and the activation energies from temperature dependent voltage-clamp experiments on frog oocytes expressing human Cx26. To analyse the effect of mutations on the temperature-dependent dye transport activity, the channel behaviour in HeLa cells expressing Cx26WT or mutants described recently (L90P, F161S, R184P)^25^ was measured optically by a microarray-based technique or into prepared microsomes. Furthermore, we tested the temperature-dependent activity on purified and reconstituted proteins (Cx26WT and Cx26K188N). With these conditions, we could screen not only the activity of connexin hemichannels and gap junctions, but we could also demonstrate that single mutations influence the temperature-sensitivity drastically.

## Results

### Identification of temperature-induced characteristics of macroscopic currents mediated by the human hemichannel hCx26

Temperature-dependent opening of the hemichannel hCx26 was first studied by two electrode voltage-clamp (TEVC) measurements under controlled temperature after heterologous expression of the hCx26 gene in potential. This post-pulse was required to ensure a complete closure of open connexin hemichannels. Applying a voltage of −80 mV caused deactivating currents of connexin hemichannels, whereas positive voltages above +10 mV activate outward currents. These outward currents initiated by hCx26 hemichannel opening increased clearly when temperature was increased above 23 °C as shown previously^33^. For comparison reason the data at + 40 mV are shown at different temperature.

**Figure 1:**
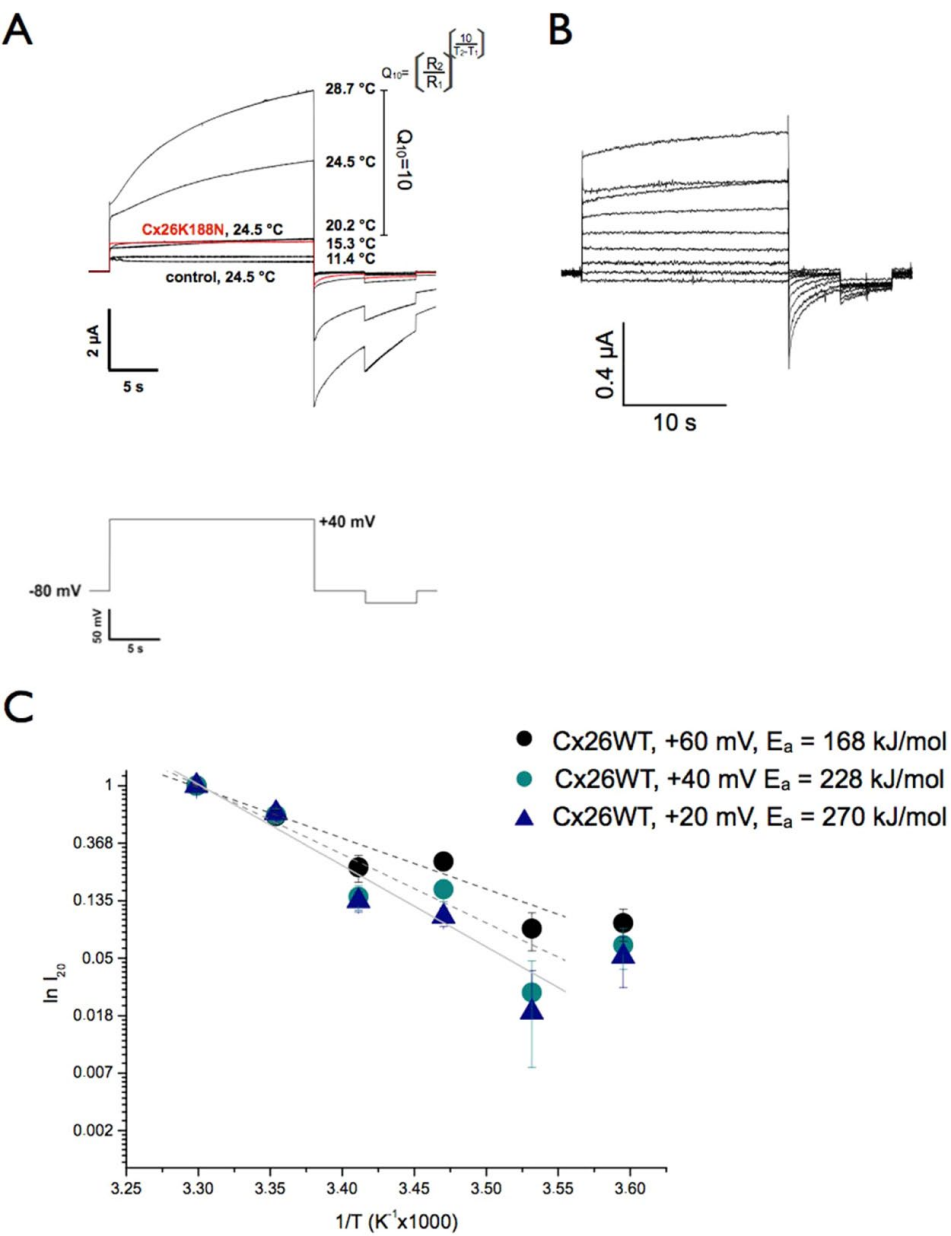
Effect of temperature on hCx26 hemichannel opening measured by TEVC. (**A**) Representative current traces are shown for hCx26 expressing oocytes injected with hCx26 cRNA and antisense Cx38 cRNA at indicated temperatures without external Ca^2+^. Control oocytes were injected with antisense Cx38 cRNA only. Current trace of hCx26 expressing oocyte at 11.4 °C (number of oocytes (n) = 6, number of measurements (m) = 7), at 15.3 °C (n = 6, m = 16), at 20.2 °C (n = 8, m = 13), at 24.5 °C (n = 9, m = 26), at 28.7 °C (n = 14, m = 33); current trace of control oocyte at 24.5 °C (n = 5, m = 6) and current trace of Cx26K188N at 24.5 °C (n = 15, m = 27) in red. Pulse protocol is shown below. From a constant holding potential of −80 mV, a potential of + 40 mV (I_10_) was applied for 20 s (I_20_). After repolarization (I_deact_) of the oocyte at a holding potential of −80 mV for 5 s a second pulse to −100 mV was applied. After 5 s, the cells were clamped at the holding potential (–80 mV) for the following 30 s. (**B**) Effect of temperature on hCx26K188N mutant activity measured by TEVC. Representative current traces are shown for hCx26K188N expressing oocytes injected with hCx26K188N cRNA and antisense Cx38 cRNA at indicated temperatures without external Ca^2+^ at 24.5 °C. Pulse protocol and experimental handling as shown in **A**. (**C**) Calculation of activation energies by Arrhenius plot given current activities (ln I_20_) as a function of temperature (1/T). The slopes of the linear function was analysed for + 20 mV (blue triangle), +40 mV (green circles) and + 60 mV (black circles).

The voltage-dependent activation resulted in an outward current composed of a biphasic mode with a fast initial rate and a slow rate (after 10 ms). Currents obtained at the indicated positions (I_10_, I_20_ and I_deact_) of the applied + 40 mV pulse (Fig. 1A) for hCx26 expressing oocytes and control cells injected with antisense Cx38 are compared in the current-temperature (I-T) plot (Supplementary Fig. S1). The temperature effect on the deactivating currents gives the same temperature dependency (Fig. S1, lower right).

Our conclusion is that the hemichannel requires an increased energy for the fully open channel. It is unknown whether the difference between the cold and warm states correlates with a closed-to-open transition since it could be that the ion passage is only reduced. As shown by Zonta before the hemichannel pore widening is influenced by Ca^2+^ ^35^. Ca^2+^ binding sites have been identified in mutations with clinical background or have been interpreted as Ca^2+^ sensor^36–39^. However, we have shown before that the Ca^2+^ blockade was not influenced by temperature^33^. Based on results obtained from protein crystallization experiments there is a cross-transmembrane domain interaction within this network^40^. The conformational change from the closed to the open channel is mediated depending on the presence of Ca^2+^. The interaction involves the amino acids K188 and E47, which are forming in the parahelical loop together with G45 and E42, a Ca^2+^ binding site of Cx26. In the work of Bennet was shown if Ca^2+^ is absent, E47 rotates at 90° and moves 7 Å away from the Ca^2+^ coordination conformation^40^. The carboxyl group of E47 is stabilized by a salt bridge with the ε-amino group of K188. Since the interaction between K188 and E47 could be critical, it suggests that a short change in the distance can affect the passage of ions. To improve this, a mutant of Cx26K188N was tested in frog oocytes as previously shown (Fig. 1A,B). It was not possible to activate Cx26K188N channel in response to the temperature as observed for the wild type Cx26WT channel and no significant outward currents were observed at temperature above 23 °C as observed for Cx26WT.

The data exhibit for the wtCx26 a Q_10_ of 10 in the measured temperature-range of 20 °C to 28.7 °C and a significant increase of I_20_ for temperatures above 20 °C (Fig. 1A). Analysing the currents after 20 s mediated by hCx26 as a function of the temperature (1/T) at different voltages reveal that an activation energy (E_a_) of 168 kJ/mol at +60 mV, 228 kJ/mol at +40 mV and 270 kJ/mol at +20 mV are required for opening (Figs 1C, S2). Enhancing the voltage for 40 mV at 29 °C resulted in a change of the activation energy of around 100 kJ/mol which are required for a fully open hemichannel.

### Development of a microarray based analysis of mutational effects on temperature-dependent Cx26 activity

**Figure 2:**
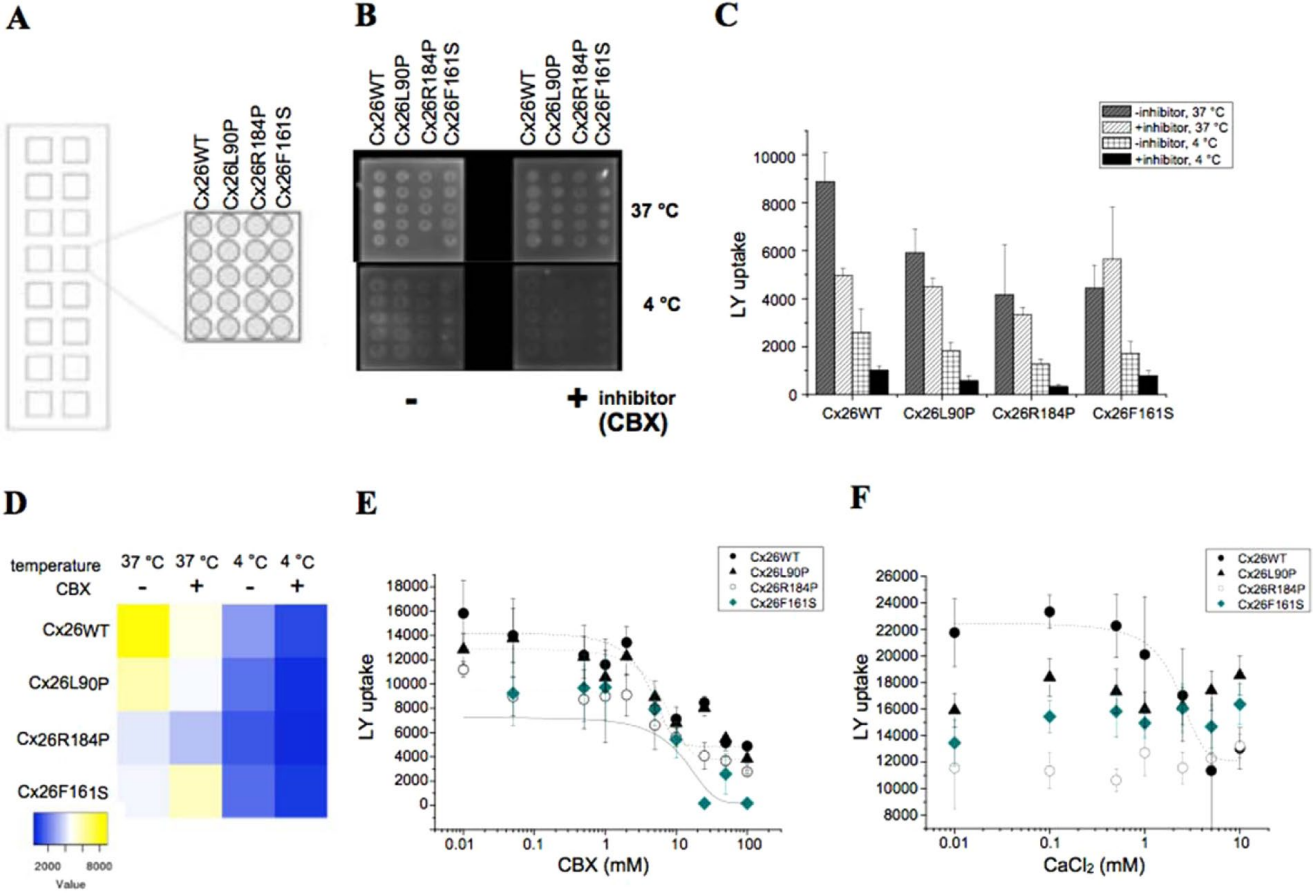
HeLa cells expressing Cx26WT and Cx26 mutants Cx26L90P, Cx26R184P and Cx26F161S for Lucifer Yellow uptake experiment. (**A**) Assignment of the cell based microarray. (**B**) Single pads of a microarray with fluorescence signal of LY at 4 °C and 37 °C with or without CBX (0.4 mM). Signal intensities are given as bar histogram as the mean ± s.d. (**C**) or heat map (**D**). Estimation of the dose-responsive activity of CBX on Cx26. (**E**) Fluorescence signal of LY at 4 °C and 37 °C with or without CBX (0–100 mM) was measured on spotted HeLa cells (scheme Fig. 1). LY uptake was monitored by fluorescence intensity of LY uptake given as a function of the CBX concentration fitted by a dose-responsive curve gave a calculated EC_50_ value of ~3 mM obtained from LY intensities of five spots given as the mean ± sd (**F**). The LY uptake was tested in presence of external increased Ca^2+^ concentrations (0–10 mM), the fluorescence intensity of LY uptake is given as a function of the external Ca^2+^ concentration fitted by a dose-responsive curve with a calculated EC_50_ value of 2 mM. Following fitting parameters with y = A1 + (A2-A1)/(1 + 10^((LOG(x_0_)-x)*p) with A1 at the highest concentration and A2 without compound at a variable Hill slope using LY intensities of five spots given as the mean ± s.d.

Several studies have shown that dyes like Lucifer Yellow (LY) and other small molecules are transported through gap junctions and connexons^30,31^. Since it is possible that there are different environmental influences between frog oocytes and human cells or recombinant-expressed proteins, the uptake of LY into HeLa cells expressing Cx26 and the effect of mutations on the channel activity was tested. We used a novel microarray based technique, which allows for the simultaneous monitoring of different mutations on dye transport mediated by Cx26. HeLa cells expressing Cx26 and mutants of Cx26 (Cx26L90, Cx26F161S and Cx26R184P) were grown in media washed in PBS and adjusted to a defined cell concentration before spotting, as described^25^. The cells were transferred into a Gesim Nano-Plotter NP2.1 and spotted onto nitrocellulose-coated microarray. After blocking the surface with BSA the cells were incubated with buffer containing LY. After 15 mins the cells were washed three times with buffer to remove external dye and fluorescence on the microarrays was analysed. A typical spotting profile is shown in Fig. 2A. In the first column, HeLa cells expressing Cx26WT were spotted with 5 replica spots in one column for statistical analysis; the following three columns contained HeLa cells expressing Cx26L90, Cx26F161S and Cx26R184P, respectively. The LY uptake into spotted HeLa cells expressing Cx26WT or Cx26 mutants on microarray visualized as the LY fluorescence is shown in Fig. 2B, with only minor LY uptake mediated by Cx26 mutants.

Figure 2C shows that carbenoxolone (CBX), a connexin inhibitor, is able to block the uptake by Cx26WT and also confirms that no LY uptake was observed at 4 °C. This is additionally illustrated by a heat map using blue corresponding with no activity and yellow corresponding with uptake activity (Fig. 2D). With the microarray assay we can monitor Cx26 mutants in parallel and observe that LY uptake was mediated by Cx26WT temperature-dependent. The treatment of HeLa cells expressing Cx26WT with CBX reduced the LY uptake in a dose responsive manner giving an EC_50_ value of ~3 mM, (Fig. 2E), whereas the cells expressing the mutants Cx26L90P and Cx26R184P have a less sensitive CBX background activity, which is not found in the Cx26F161S mutant. The LY uptake mediated by cells expressing Cx26WT is more sensitive to increasing external Ca^2+^ concentration (EC50 value~2 mM) for the LY uptake (Fig. 2F). Furthermore, it was suggested that open Cx26 channel mediate the passage of small molecules like anti Hsp90 inhibitors like 17AAG or radicicol. Therefore, a difference in the vitality between HeLa cells with Cx26wt and Cx26 mutant should be visible. In presence of EDTA and 37 °C the vitality of Cx26wt HeLa cells was reduced with 17AAG but not with radicicol and as suggested not for HeLa cells with the mutant Cx26L90P. HeLa cells with the mutant Cx26R184P showed a minor vitality than the other cells and had also a weaker reduction in the vitality whereas on HeLa Cx26F161S cells no reduction in the vitality was detected (Fig. S5). In the presence of 5 mM EDTA, no vitality of all HeLa cell types was observed. The data show that an anti Hsp90 activity is mediated due to the activity of open Cx26 channels.

**Figure 3:**
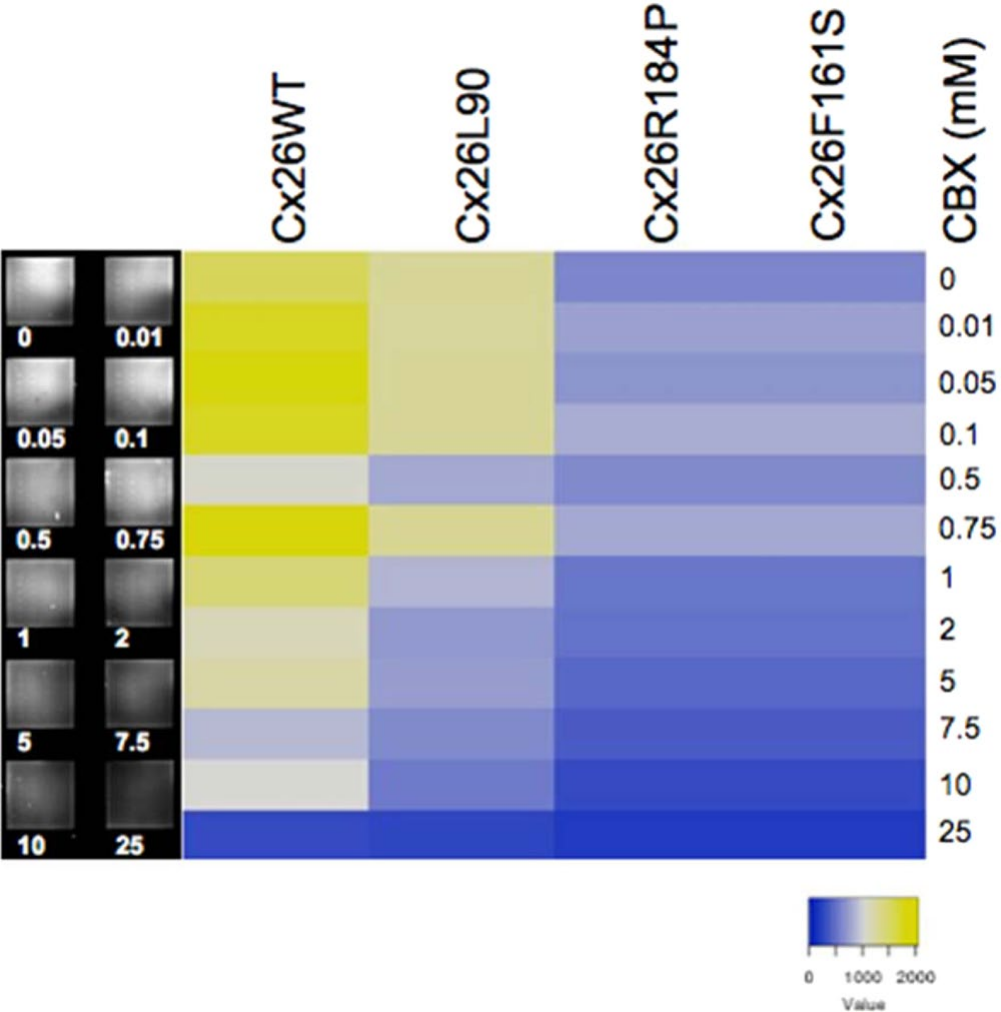
Fluorescence signal of LY uptake was measured on spotted microsomes obtained from HeLa cells expressing Cx26WT and Cx26 mutants Cx26L90P, Cx26R184P and Cx26F161S at 37 °C without or increasing CBX concentrations (0–25 mM). Signal intensities are given as heat map (right), microarray (left).

The LY flux assay was then performed as before but with microsomes isolated from HeLa cells (microsome flux assay (MFA)), to determine whether existing connexosomes could mediate uptake and therefore not be correctly trafficked to the plasma membrane. Microsomes isolated from HeLa cells expressing Cx26WT or mutants were spotted in columns of ten spots onto the NC surface. After transfer into an incubation chamber, single pads of the microarray were pre-incubated with buffer containing different concentrations of CBX at the indicated temperature and subsequently for uptake with LY at 4 °C and 37 °C. The signal intensity of LY uptake into microsomes is much weaker at 37 °C and at 4 °C no LY uptake was observed (data not shown). The LY uptake mediated by vesicles at 37 °C was for Cx26WT sensitive for CBX in a dose-responsive manner, the mutants showed much weaker or no uptake activity (Fig. 3). Here we show that the measurement of temperature-dependent connexon activity can be performed by microarray-based technique on cells as well restored from vesicles.

### Mutational influence of the temperature-dependent Cx26 hemichannel activity on the protein level

Recently, the equilibrated structure of hCx26 described first by Kwon *et al*. (2011, 2012) gave the hint suggested that several interactions, including glutamic acid position 47 (E47) with arginine position 184 (R184), E47 with arginine position 75 (R75), and aspartic acid position 46 (D46) with R184 and lysine position 188 (K188), are broken and reformed with only the thermal energy provided by the simulation (0.62 kcal/mol at 310 K)^41,42^. Several positions were conserved for folding, trafficking or other reasons. It was suggested before that the mutations in Cx26L90P and Cx26R184P influence trafficking^25,27^.

The aim was to analyse the function on the purified protein directly to exclude any internal cellular effects, which may also result in a loss of temperature-dependent activity. It is suggested that the position of K188 is highly conserved whereas the reason is unknown. It could be that the exchange in Cx26K188N influence trafficking in frog oocytes due to misfolding or functional consequences. Therefore the mutant construct with one amino acid exchange in K188 to N188 was generated for protein expression in *E*. *coli* to studying the temperature effect directly on the protein.

After removal of the N-terminal tag by SUMO protease and addition of lipid (POPC) in a 1:1 (w/w) lipid to protein ratio, the monomeric channel protein formed hexamers. The undigested form was running at 29–30 kDa, whereas after tag removal the monomeric band was identified at ~26 kDa and the hexameric form was at ~160 kDa in SDS-PAGE (Fig. S3). We can see that Cx26K188N formed stable hexamers after reconstitution in liposomes.

**Figure 4:**
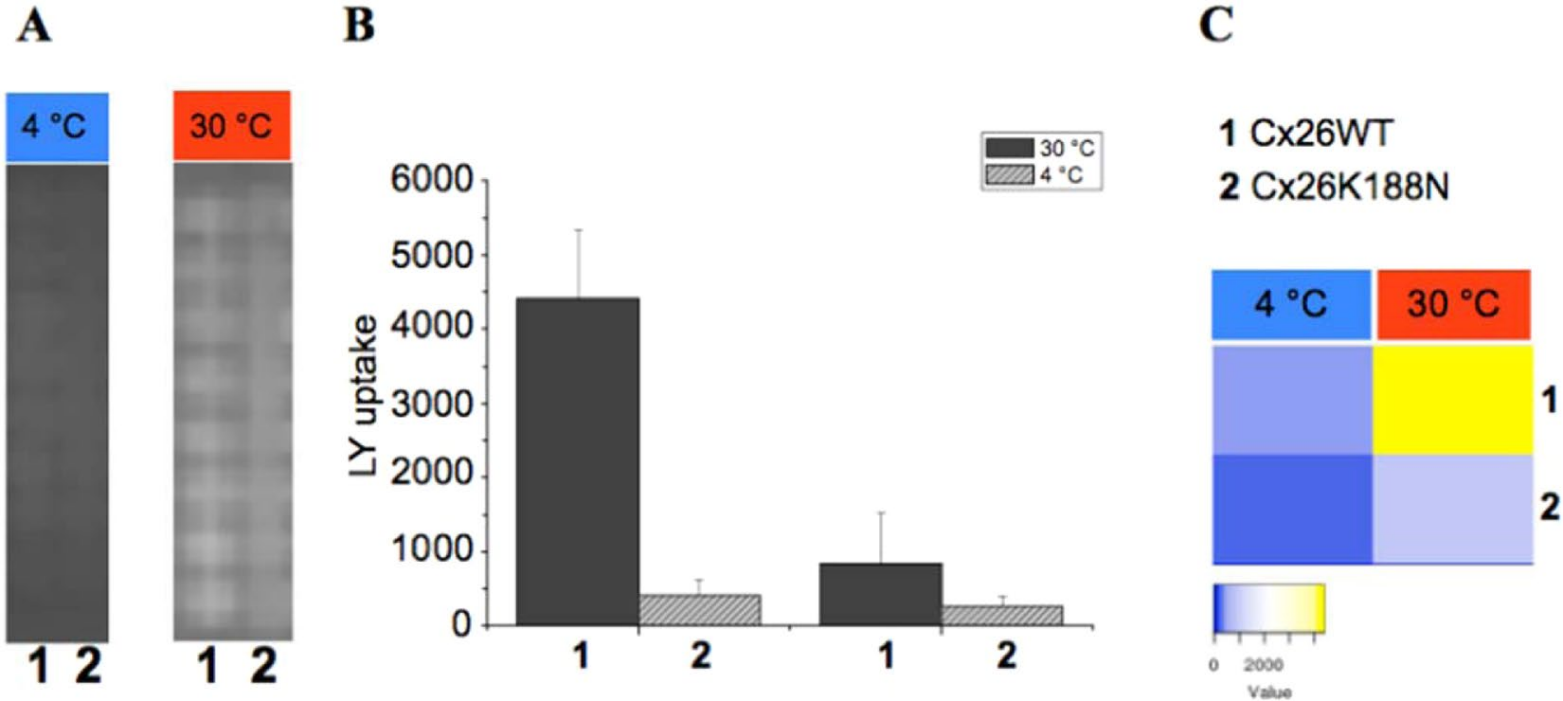
Microarray based LY uptake by purified and reconstituted Cx26WT (1) Cx26K188N (2) into liposomes (**A**) Single pads of a microarray with fluorescence signal of LY at 4 °C and 30 °C. Signal intensities are given as bar histogram (**B**, middle) or heat map (**C**, right).

The refolded purified hemichannels were reconstituted into liposomes. Afterwards the liposomes containing reconstituted proteins were spotted on the NC-microarrays and the temperature dependent LY uptake Cx26WT and mutant was analysed. Figure 4 shows the microarray-based temperature-dependent LY uptake experiment mediated by Cx26WT and Cx26K188N. At low temperature at 4 °C (Fig. 4A–C,) LY fluorescence has background activity only, but is increased at 30 °C. In contrast, the LY uptake was drastically reduced in Cx26K188N.

**Figure 5:**
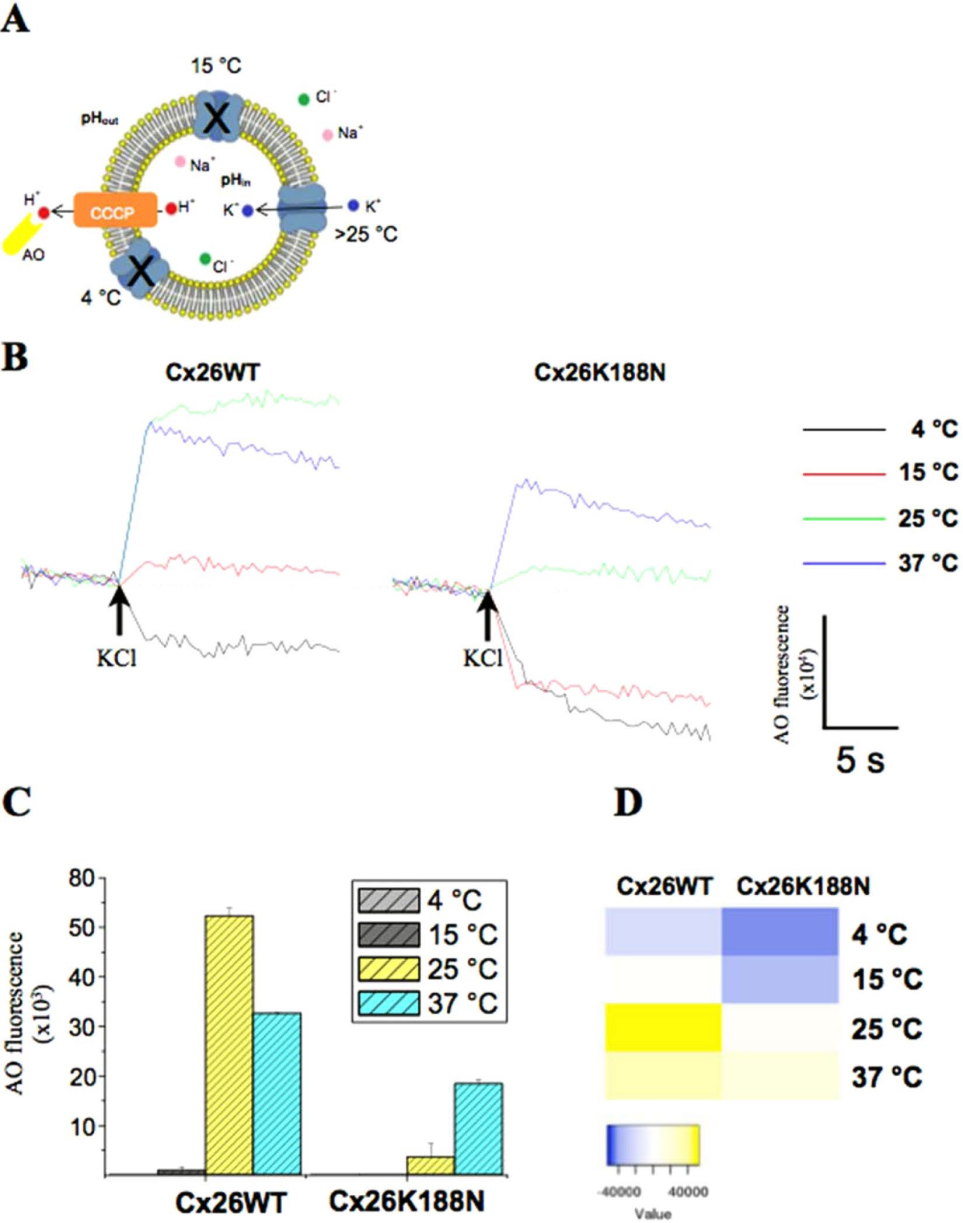
Temperature-dependent LFA for connexin26. (**A**) Scheme of LFA according to Su *et al*., (2016)^43^. Thawed vesicles equilibrated at 37 °C are diluted into 20 mM Tris-HCl, pH 8.0, 300 mM NaCl, 1 mM EDTA 6.5 μM AO (acridine orange), 16 μM CCCP (cyanide m-chlorophenylhydrazone). Ion channel K^+^ uptake activities are initiated by the injection of a buffered potassium solution (20 mM Tris-HCl, pH 8.0, 1 M KCl) induces a gradient on the vesicle membrane. The H^+^ efflux is monitored by the fluorescence increase of a fluorescent dye, acridine orange. (**B**) Normalized representative kinetics of reconstituted Cx26WT and Cx26K188N. Channel activities induced by injection of potassium chloride (arrow) at different temperature (black traces for 4 °C, red for 15 °C, green for 25 °C and blue for 37 °C) using for excitation 485 nm wavelength and 530 nm wavelength for emission. After 10 s a volume of 10 µL 20 mM Tris-HCl, pH 8.0, 1 M KCl (arrows) was injected into the running kinetic and observed for additional 15 s. For comparison plots of activities given as histogram (**C**) and representative as heat map (**D**).

To study the temperature dependent kinetic of Cx26WT and Cx26K188N, the liposomes were transferred to the LFA and the temperature-dependent kinetic was monitored. A similar method was used by Su *et al*. recently for the identification of K^+^ channel activities^43^. The scheme represents the components which have been reconstituted with acridine orange (AO), CCCP (cyanide m-chlorophenylhydrazone), lipid vesicles, protein using internal NaCl pH 7 and external potassium chloride at pH 8. Ion channel K^+^ uptake activities are initiated by the injection of a buffered potassium solution (20 mM Tris-HCl, pH 8.0, 1 M KCl) inducing a gradient on the vesicle membrane. The H^+^ efflux is monitored by the fluorescence increase of the fluorescent dye (AO) (Fig. 5B). The reconstituted Cx26WT hemichannels mediated a rapid temperature-dependent dye release activity above 15 °C after injection of potassium chloride, whereas the K188N mutant had minor activity only at 37 °C (Fig. 5B). It could be that gradients in liposomes not stable at 37 °C. Liposomes without protein as a control showed no increase in the fluorescence (Fig. S4), but the signal intensity decreased after injection as observed for low temperature activity with the reconstituted Cx26 connexins (Fig. 5B). We concluded that with the injection of potassium chloride the uptake activity by the temperature-dependent activity of the connexins induced the proton release by CCCP accompanied with release of AO (Scheme Fig. 5A). The temperature-dependent transport activity is not only selective for dye molecules such as LY, but the passage for potassium is also blocked at low temperature. Furthermore, the data indicate indeed that it is possible to refold mutated Cx26K188N to hexamers *in vitro* as observed in Fig. S3, but Cx26K188N was neither active after expression in oocytes nor as purified protein after reconstitution into liposomes like the Cx26WT. Other results have shown before, that the position K188 is conserved^27^. Furthermore, the hexameric mutant channel showed only a limited activity at higher temperature (Fig. 5).

## Discussion

Microarrays are a promising tool for global analytical approaches with reduced consumption of time, analytes and reagents. Here, new microarray formats were developed to monitor temperature-dependent hemichannel activity of Cx26 in cells, vesicles or liposomes. This microarray technique enabled the simultaneous comparison of Cx26 hemichannel activity of wild type and mutants. Within this article we demonstrate that microarrays are a promising tool for parallelized studies using cells and vesicle/liposomes to examine transport processes. Therefore in future the technique can help to investigate mutational effects simultaneous. Due to the highly miniaturised plotting technique using nanoliter volumes, a volume of 50 µL is sufficient for hundreds to thousands of microarrays and helps to reduce the costs.

As predicted from previous results, Cx26 hemichannels are inactive at a non-physiological temperature below 23 °C^33^. This is not observed for Cx46^44^. How other connexin hemichannels react under temperature changes has not been investigated yet. Mutations in Cx26 influence the hemichannel activity drastically. This can result from trafficking accompanied with incorrect folding and lower physical presence, or functional effects during formation of gap junction or gating. Considering the trafficking and folding effects separately, we can show that K188N is a correctly folded hexameric hemichannel when synthesized in *E*. *coli*, but shows no or weak temperature sensitivity and is not active as the wild-type channel. Structural studies have shown that the closed form of the Cx26 gap junction structure is occupied with Ca^2+^ in the bound state whereas the Ca^2+^ free state is accepted as the open form^40^.

We have shown that a single point mutation in K188N compensated for the close-to-open transition and concluded that the activation energy of 270 kJ/mol at +20 mV or even higher voltages is required for the side chain movement of E47 and formation of the salt bridge with K188^40^. The mutation in the position K188N was used since it could be required for the recently identified Ca^2+^ binding site E42/E47/G45. In addition, our electrophysiological and optical measurements show that the response from closed to open state is rapid. We suggest that it could be mediated by a rapid positional change of the E47 towards K188 which then becomes stabilized. Other larger structural transitions required for the fully open channel may take a longer time. Therefore, it is suggested that the distances between the side chains of K188 and E47 and the corresponding shift of E47 towards K188 are important for the thermal induced stabilization/destabilization forming a stable salt bridge. As shown before, Ca^2+^ blocking could not be compensated by an increase in temperature, since the binding energy for Ca^2+^ is much higher than the formed salt bridge between ε-K188 and E47^40–42,45^. The exchange of K188 to N188 leads to an additional charge where E47 is unable contact the shorter N188. At the entrance of the alpha helix TM4, K188 is a part of a subunit-spanning electrostatic network, which contains the amino acids D46, E47, R75, R184, D42, E187 and S72. The latter connects the respective subunits of the hemichannels and represents relevant positions in the loop gating region. The open state of the channel is stabilized by multiple weak electrostatic interactions of this network. Furthermore, it is believed that interference with the electrostatic network could affect the stabilization of the channel due to this network. The interactions within the network, i.a. also the interaction of K188 with E47, e.g., already can formed by increased temperature above 23 °C. Highly structural resolution studies of other gap junction channels demonstrated that despite having a similar architecture, differences in the form of the water filled funnel of the channel exist^32^. However, relevant structural differences between Cx46/50 and Cx26 are identified. The structural comparison shows that the Cx26 has a disordered structure in the NTH domain and acetylation is relevant for the pronounced selectivity. These findings are in agreement with recent molecular simulation studies on Cx26 where the temperature changed the diameter of the channel tunnel at the cytoplasmic side by widening the C-terminal and cytoplasmic domains, however a highly flexible dynamic NTH structure was also observed^46^. As analysed previously by a MD simulation study, the structure of a fully open hemichannel can be different than an open gap junction channel. The physiological role of the thermosensitivity in the context of Cx26 is unknown. Interestingly, this characteristic is not present in other hemichannels, e.g., Cx46^44^. A novel role of hemichannels have been demonstrated recently by the analysis of mutations regarding Cx26-linked diseases and how gene mutations lead to hearing loss or to hearing loss combined with skin disorders of varying severity^36^. From several mutations in NTH/TM1 in Cx26 it is known that the position D50N exhibits a reduced Ca^2+^sensitivity^36–38^. As shown by others, the effect of mutations can have medicinal consequences, since ATP, IP_3_ or interleukin signalling is modulated and associated with a variety of inflammatory processes and tumour development^47,48^.

Thermo-sensitivity has been first described for thermo responsive protein (TRP) channels, which are involved in thermo-reception, mechano-reception and nociception across a wide range of environmental stimuli^49^. Temperature modulates aging, development, reproduction, mating, and circadian rhythm^50^. Modest and sustained reduction of core body temperature has anti-aging effects and prolonged life span in mice^51^. The effects observed by hypothermia are similar to the effects observed with caloric restriction^50^. Thus, metabolic changes might be induced that are favourable and can delay aging. As has been shown for nearly all connexins, the turnover of the Cx26 is very rapid leading to an exchange of all gap junctions within 24 hours. Such a high metabolic state may be dangerous in conjunction with hazardous low or too high temperatures, especially in the inner ear where hearing takes place in the presence of a stimulus not allowing a deliberate control of the process. The thermo-sensitivity of the channel might be considered an endogenous protective mechanism leading to closure of hemichannels and down regulation of the high metabolic state at hazardous low temperatures. Such low temperatures may be of relevance in cold ischemia in organ transplantation (e.g., heart) and for the skin^51^. Cx26 was recently identified in cardiomyocytes^52^. This may be relevant since connexins are involved in electrical and chemical synchronization of cells in organs and tissues^53,54^. Recently, it was shown that Cx45 gap junctions gave faster deactivation rates at higher temperatures than at lower temperatures^55^. This uncoupling could play an important role in regulating action potential propagation speed in Cx45 enriched nodal cells in the heart. Inhibition of endothelial retinoid uptake by 5′-methoxyleoligin, a compound from Edelweiss, had cardioprotective properties^56^. This example indicates that permeability in both direction via hemichannels has an relevant physiological impact for the uptake of small molecules and is not only a limited release of small molecules such as ATP^57^. This also suggests that small molecules toxic for cells can also passage through the open hemichannels into the cells and that even the opposite uptake/release pathways require regulation. Since Cx26 is closed at low hazardous temperature, no uptake of toxic molecules is supported and perhaps protects the cell. This is in line with recent reports on therapeutic hypothermia being able to protect the cochlea from noise-induced damage^58,59^.

We have monitored Cx26 hemichannel activity when the external Ca^2+^ concentration is low (<1 mM) by electrophysiological measurements and performed parallel analyses on cell/liposome using a microarray-based technique. In future, this technique will help to analyse mutational effects or compound transport targeting the channel folding or activity. We identified and confirmed with this technique on the cold to warm activation of a temperature-dependent process, which requires a thermal sensor for the activation. One position relevant for this process is K188. The temperature sensitivity of Cx26 has not been widely studied and is only evident under focussed experimental or physiological conditions. We propose that the temperature-dependent deactivation of hemichannels could be an osmotic regulation mechanism to protect cells from loss of colloids or hinder uptake of toxic molecules. In both cases, a controlled thermal regulation is essential for the skin and other organs.

## Methods

### Materials

POPC (1-palmitoyl-2-oleoyl-sn-glycero-3-phosphocholine) obtained from Avanti Polar Lipids, Inc. (Alabaster, Alabama, USA). Nickel or Cobalt-chelating resin purchased from Jenabioscience (Jena, Germany), OG (n-octyl ß-D-glucopyranoside) from Glycon Biochemicals GmbH, (Luckenwalde, Germany) and Topo cloning kits from Life Technologies, GmbH, (Darmstadt, Germany). The *E*. *coli* strain TOP10 and BL21plys (Invitrogen) were used to host the plasmids pGEMHE and pETSUMO containing the hCx26 gene. The vector pGEMHE with Cx26WT, Cx26K188N was used for cRNA synthesis^33^. *E*. *coli* cells were cultivated in LB medium using ampicillin as selection marker (100 µg ml^−1^) at 37 °C.

### Oocyte expression and TEVC measurements at different temperatures

Temperature controlled oocyte measurements were performed as described previously^33^. Buffer composition and injected cRNA concentration adjusted for the expression of hCx26 as described before^60^. A solution of 23 nl containing hCx26-cRNA (2 μg μl^−1^) and anti-Cx38 (0.4 μg μl^−1^) was injected into *Xenopus laevis* oocytes. Control oocytes were injected with 23 nl of a solution containing only anti-Cx38 (5 h 23ation contain CTG TCC ACA CAG-3′; 0.4 μg μl^−1^). For expression, oocytes were conserved for at least 24 h at 16 °C in modified Barth medium (88 mM NaCl, 1 mM KCl, 2.4 mM NaHCO_3_, 0.33 mM Ca(NO_3_)_2_, 0.41 mM CaCl_2_, 0.82 mM MgCl_2_, and 15 mM HEPES at pH 7.4). Voltage-clamp experiments were performed as described previously^61^ using a nominal Ca^2+^-free bath solution composed of (88 mM NaCl, 1 mM KCl, 2.4 mM NaHCO_3_, 0.82 mM MgCl_2_, and 15 mM HEPES at pH 7.4). Temperature-induced hemichannel activity was measured with TEVC^33^. Briefly, oocytes were clamped at −80 mV and currents were evoked by a voltage change from −100 mV to + 60 mV in alternating steps of 20 mV applied for 20 s. Currents obtained after 20 s are named I_20_. After repolarisation of the oocyte at a holding potential of −80 mV for 5 s a post pulse to −100 mV followed and the cells were clamped after additional 5 s to the holding potential of −80 mV. The temperature of the bath solution was adjusted during the measurements by a flow chamber, which was mounted under the cuvette and connected to a temperature controlled heat supply. A temperature sensor in the vicinity of the oocyte was employed to measure the actual temperature of the bath solution at the beginning and end of each voltage protocol. The data are expressed as mean ± standard error of mean (sem) and the descriptive statistical analysis was performed using OriginPro 9.1 (OriginLab Cooperation). The activation/deactivation rates were obtained from exponential fits 2nd order (Origin 7.5).

### Calculation of Q10 and activation energy (E_a_)

As described by Steffens *et al*., 2008^33^ the voltage pulse protocol was used: starting at a constant holding potential of −80 mV voltages from −100 mV to + 60 mV were applied for 20 s in steps of 20 mV. 5 s after returning to holding potential of − 80 mV a post pulse to − 100 mV for 5 s followed before returning to the holding potential of −80 mV. Between two test pulses, a 30-s resting phase at the holding potential was followed. I_20_ denotes the interval at the end of the 20-s conditioning voltage pulses where the amplitude of the evoked currents measured (I_20_). I_10_ denotes the interval at the end of the 10-s conditioning voltage pulses where the amplitude of the evoked currents measured (I_10_).

Q_10_ is used to describe the temperature dependence of a process. It is a unit less temperature coefficient and is a measure of the rate of change of a biological or chemical system as a consequence of increasing the temperature by 10 °C. Q_10_ calculations were performed by Q_10_ = (R_2_/R_1_)^10^ ^°C/(T^_2_^-T^_1_^)^; with R_1_ is the rate which represent the ion flux in µA after 20 s at temperature T_1_ in Celsius degrees, whereas the rate R_2_ is obtained at the higher temperature T_2_.

The activation energy (E_a_), was calculated from linear slopes from semi logarithm plot using normalized currents vs temperature 1/T^62^. Therefore normalized currents at I_20_ were obtained and the calculations were performed according to I_20_ = A e^−Ea/RT^; with current at 20 s (I_20_) Arrhenius constant A activation energy E_a_ (kJ/mole), R the gas constant (8.31447 J/mole K) and T temperature (K).

### HeLa cell cultivation for microarray based Lucifer Yellow (LY) Uptake assay (LYUA)

HeLa cells expressing Cx26WT or mutated Cx26 (L90P, F161S, R184P) were cultivated for 3 days at 37 °C in a humidified environment with 5% CO_2_. For microarray spotting, cells were harvested using trypsin (incl. 0.02% EDTA) and concentrated in PBS-medium to 4 × 10^6^ cells/mL. The cell suspension was mixed immediately before the printing. Afterwards the cells were printed onto a nitrocellulose (NC) coated microarray utilizing a contact-free piezoelectric Nano-Plotter (NP_2.1, GeSim) as described before using around 80 droplets per spot and spotting parameters 100 Hz, 180 µs, 100 V and pulse width of 50, one droplet was equal to the volume of 0.35 nL, and the maximum immobilized suspension volume was 1.2 µL^63^. After spotting the microarray slides were inserted into a chamber (Nexterion) for separate liquid incubation of the 16 pads at a volume of 50 µL, respectively. The microarrays were incubated with DMEM (Dulbecco’s Modified Eagle Medium)-containing 10% FBS to cover unspecific sites on the NC. For inhibitor tests the cells were preincubated with inhibitors at indicated concentrations. Next, the pads were incubated with EM buffer (120 mM NaCl, 7 mM KCl, 0.8 mM MgCl_2_, 5 mM glucose, and 25 mM Hepes at pH 7.3) containing 2% Lucifer Yellow for 15 min at indicated temperature and free dye were removed by three exchanges with EM buffer and incubated 10 min at indicated temperature, respectively. The supernatant were removed and the fluorescence signals were analyzed by use of a Laser Scanner (SensoSpot® Fluorescence and Colorimetry Microarray Analyzer, Miltenyi Biotec Company, Germany) using blue illumination (410–480 nm wavelenght) for 5 s and fluorescent signals were calculated with providers pre-installed array analysis software (Sensospot). Data were analyzed and performed using OriginPro 9.1 (OriginLab Cooperation) and for heat map presentation heatmapper was used.

### Preparation of microsomes for microarray flux assay (MFA)

Cultivated HeLa cells (4 × 10^6^ cells/mL) with Cx26WT or mutated Cx26 were concentrated and resuspended in a PBS buffer containing protease inhibitor. The suspension was transferred three times through a 10 cm long injection needle. Afterwards the homogenate was centrifuged at 10,000 × g for 10 min at 4 °C and the supernatant sedimented at 50,000 × g for 30 min at 4 °C. Next the sediment was resuspended in 100 µL TBS. The vesicles were centrifugated for 30 min at 5,000 × g or filtrated through 0.2 µm filter and spotted as described before onto the nitrocellulose of a microarray utilizing a contact-free piezoelectric nanoplotter (NanoplotterNP2.1, GeSim) using around 20 droplets per spot and spotting parameters 100 Hz, 50 µs and 80 V one droplet was equal to the volume of 0.8 nL. The treatment and readout protocol for LY uptake is the same as used before.

### Recombinant synthesis of channel constructs

The codon usage of the human Cx26WT sequence was adapted to that of *E*. *coli*. The Cx26K188N was generated by PCR/DpnI technique using mutagenic primers (for: TCC AAC CGA GAA TAC CGT GTT TAC and rev: GTA AAC ACG GTA TTC TCG GTT GGA)^64^. Afterwards, the Cx26WT and Cx26K188N sequences were cloned into the pETSUMO vector, respectively. The corresponding constructs were synthesized in *E*. *coli* BL21DE3 cells and hCx26WT and Cx26K188N were purified by metal-chelating affinity chromatography (IMAC) as described previously^33,61^. Expression of proteins and presence of recombinantly synthesized channel proteins was improved before by immune blots. In addition the proteins expressed as described previously and purified by a cobalt-chelating resin^33,34,61^. *E*. *coli* lysates from 10–15 g cells were solubilized in 200 ml N-lauroylsarcosine (NLS) buffer, containing 1% NLS, 200 mM NaCl, 50 mM NaHPO_4_, 1.09 M glycerine and 10 mM Tris pH 9.5, supplemented with 20 µl protease inhibitor (P8849, Sigma-Aldrich). The solution was stirred on ice for 2 h and centrifuged at 30,000 × g for 1 h. The supernatant was incubated with 7 ml IMAC resin at a lower NLS concentration of 0.5% at 4 °C for 16 h. Unbound protein was removed by washing with 50 ml 0.5% NLS, 200 mM NaCl, and 10 mM Tris pH 8, and an additional washing step with 10 ml octylglucoside (OG) buffer, containing 30 mM OG, 200 mM NaCl, and 10 mM Tris pH 8. The bound hCx26 protein was eluted with 30 mM OG, 500 mM imidazole and 10 mM Tris pH 8. Elution fractions were diluted 5-fold with 200 mM NaCl, 20 mM Tris pH 8, 30 mM OG before being concentrated ten-fold with a YM 30 K concentrator (Millipore) by centrifugation at 3,000 × g followed by three more cycles of dilution and concentration. The concentration of the protein was adjusted to 1–10 mg ml^−1^. The tag of 2 mg purified hCx26 was removed in the presence of SUMO protease over night at 4 °C. After addition of POPC to the digested hCx26 oligomeric protein were separated by SEC. 16/60 chromatography. Purified and folded hCx26 was stored at a concentration of 1–5 mg ml^−1^ in buffer (10 mM Tris pH 8, 200 mM NaCl and 30 mM OG) at −80 °C.

### Reconstitution of purified Cx26 hemichannels

A lipid film of a chloroform solution containing 10 mg POPC lipid dried under nitrogen was dissolved in buffer (10 mM Tris pH 7.0, 300 mM NaCl, 1 mM EDTA). The solution was incubated at 37 °C for 30 min, sonicated and pressed through a 0.2 µm filter ten times. This suspension was incubated with 100 µg hCx26WT or Cx26K188N on ice overnight and shaked additional 1 h at room temperature. Control liposomes were prepared without channel protein. The mixture was passed through a PD10 gel filtration and the liposomes were collected at 65,000 rpm in BECKMAN rotor 100.1 at 4 °C for 30 min and sediments were resuspended in ice cooled free washing buffer (10 mM Tris-HCl pH 7.0, 300 mM NaCl, 1 mM EDTA). The liposomes were transferred for activity measurements to the LFA.

### Temperature-dependent liposome flux assay (LFA)

Temperature dependent activity of purified and reconstituted Cx26WT or mutant Cx26K188N hemichannels were analysed by liposome flux assay (LFA) using Acridine Orange (AO) for optical monitoring with minor modification according to Su *et al*., 2016^43^. Liposomes containing channel proteins were thawed in a 37 °C water bath for 30 min and briefly mixed to homogeny by gentle pipetting using around 20 µL liposomes in 96 well microplates containing 20 mM Tris-HCl, pH 8.0, 300 mM NaCl, 1 mM EDTA 6.5 μM AO, 16 μM CCCP. Before injection of a volume of 10 µL 20 mM Tris-HCl, pH 8.0, 1 M KCl to a final volume of 250 µL in the well the temperature of the plates were adjusted to the indicated temperature. The measurements were performed in a Berthold Mithras 470 multiplate reader using for the excitation of the fluorescent dye a wavelength of 485 nm and for emission a wavelength of 530 nm. Fluorescent signal was monitored every 0.5 s for every 21 repeats before injection and 31 repeats after injection. The kinetic measurements have been analysed by software OriginPro 9.1 (OriginLab Cooperation).

### Vitality assay

HeLa cells expressing Cx26WT or mutants were transferred to a 96 well microplate (Nunc) with a cell concentration of 2.5*10^5^ cells/mL and were incubated for 24 hours at 37 °C and 5% CO_2_. The compounds (17AAG, radicicol) were added with a final concentration of 5 µM and the cells were incubated for one hour in presence of 1.8 mM EDTA. The inhibitors were removed, fresh medium was added and the cells were incubated for additional 24 h. Next, the medium was removed and the cell proliferation reagent WST-1 (10%, Roche) was added and incubated for half an hour. The measurements were performed in a microplate reader (Synergy H1 Hybyid Reader, BioTek). The wavelength for measuring the absorbance of the formazan product is 450 nm. The reference wavelength is 630 nm.

## Supplementary information

Figures S1 - S5.

## Data Availability

All data concerning electrophysiological or optical measurements are stored on online server as well cell clones and Cx26 plasmids are available on Figshare as follows: Figure 1. 10.6084/m9.figshare.9787973.v1; Figure 2. 10.6084/m9.figshare.9786275.v1; Figure 3. 10.6084/m9.figshare.9786950.v1; Figure 4. 10.6084/m9.figshare.9787418.v1; Figure 5. 10.6084/m9.figshare.9787589.v1.
