# Supplementary material for: Microarray-based screening system identifies temperature-controlled activity of Connexin 26 that is distorted by mutations
Source: Sci Rep. 2019 Sep 19;9:13543. doi: 10.1038/s41598-019-49423-3 (PMC6753059; doi:10.1038/s41598-019-49423-3)
Supplement: Supplementary file 1 — Figures S1 - S5. [file 41598_2019_49423_MOESM1_ESM.docx]

**Supplementary file**

Microarray-based screening system identifies temperature-controlled activity of Connexin 26 that is distorted by mutations

Hongling Wang^1,3^, Frank Stahl^2,3^, Thomas Scheper^2,3^, Melanie Steffens^1^, Athanasia Warnecke^1^, Carsten Zeilinger^3*^


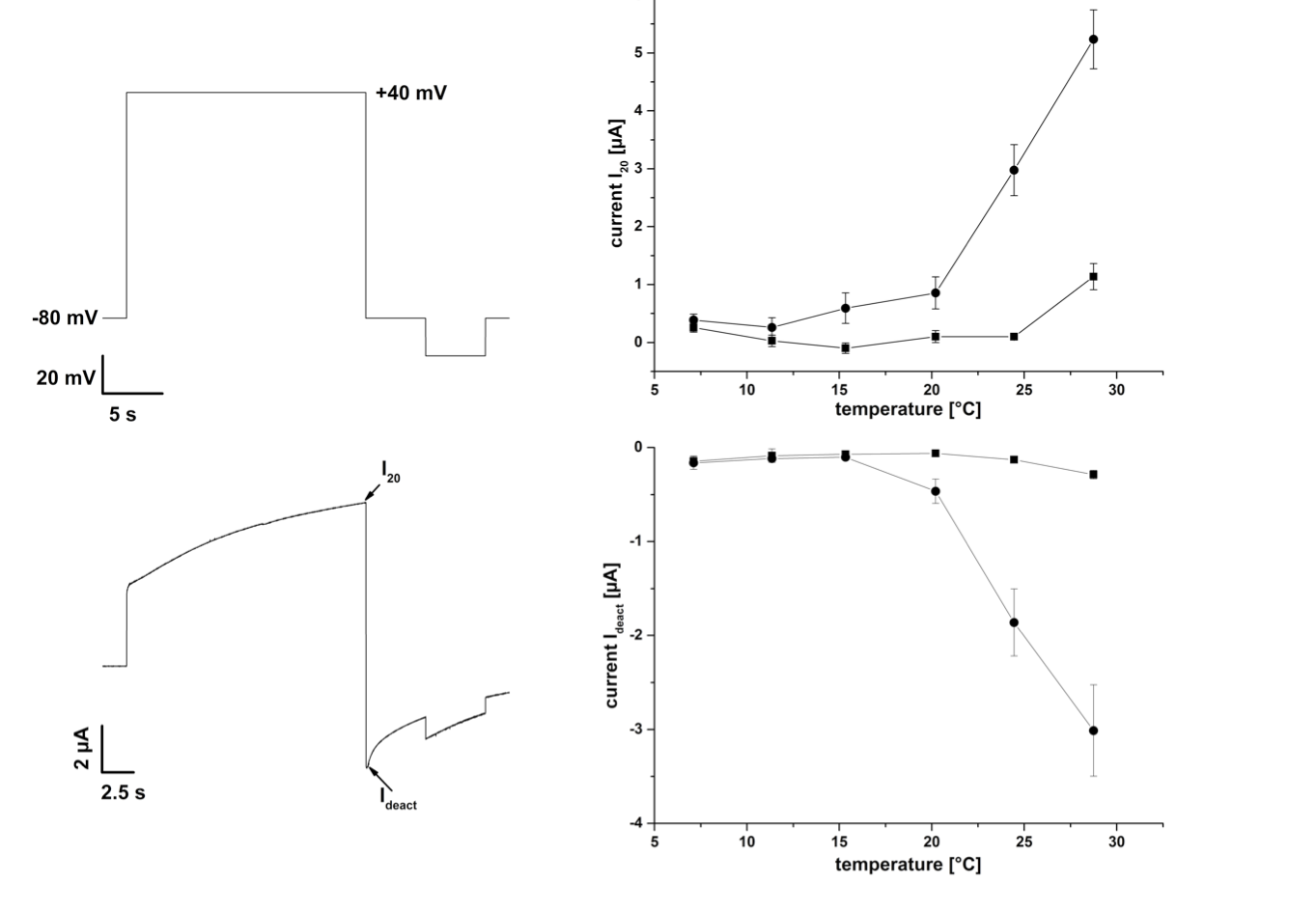


Figure S1: Current-temperature plot obtained as response to the application of a 20 s +40 mV pulse (upper right, I_20_) and return to the holding potential of ‑80 mV (lower right, I_deact_) for hCx26 expressing oocytes (solid circles) and control oocytes (solid squares). Each data point are mean ± sem for at least 5 experiments for hCx26 expressing oocytes and control oocytes (hCx26: n=6-14, m=7-33; control: n=5-9, m=6-12. Pulse protocol for (lower left) corresponding current evoked by the voltage application. The arrows indicates the positions at which the current I_20_ and I_deact_ was measured for the current / temperature plot.

Figure S2: Current-voltage relationship of hCx26 (Fig. 1A) mediating currents at different temperatures and corresponding initial I_10_ (B) and I_20_ (Fig. S1) activation/deactivation rates for hCx26. Mean ± sem of current obtained after 20 s at applied voltage. (n = oocytes and m = measurements; hCx26: n=6-14, m=7-33; control: n=5, m=6-12). The measurements were performed in presence of anti sense Cx38 and activation/deactivation rates obtained from exponential fits 2nd order, with filled squares for deactivation and filled circles for activation


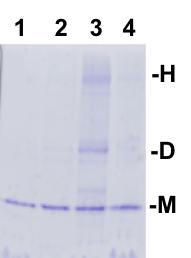


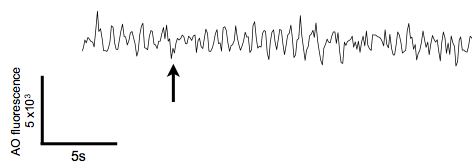
Figure S3: Purified Cx26WT and Cx26K188N protein after IMAC, SUMO protease digest and SEC16/60 purification. SDS PAGE after SEC purification. Predominant monomeric bands (M) with 1= Cx26K188N and 2= Cx26WT separated from oligomers 3=Cx26K188N and 4=Cx26WT, whereas fraction containing hexameric bands used for reconstitution. Hexameric (H) dimeric (D) and monomeric (M) bands indicated at the correct molecular mass.

Figure S4: LFA with liposomes. Thawed liposomes without protein equilibrated at 37 °C are diluted into 20 mM Tris-HCl, pH 8.0, 300 mM NaCl, 1 mM EDTA 6.5 μM AO (acridine orange), 16 μM CCCP (cyanide m-chlorophenylhydrazone). Putative transport activity are initiated by the injection of a buffered potassium solution (20 mM Tris-HCl, pH 8.0, 1M KCl indicated by an arrow) induces a gradient on the vesicle membrane. The H^+^ efflux is monitored by the fluorescence of AO.

Figure S5: Influence of anti Hsp90 inhibitors on the vitality of HeLa Cx26wt and Cx26 mutant cell types. The formazan signal at 450 nm was measured after preincubation of the cell types with anti Hsp90 inhibitors 17AAG (5 µM) or radicicol (5 µM) in presence of 1.8 mM EDTA and 37 °C^65^. The data normalised to the control of HeLa Cx26wt. Data are presented as mean ± s.d. from at least 2 independent experiments.

65. Franke, J., Eichner, S., Zeilinger, C. & Kirschning, A. Targeting heat-shock-protein 90 (Hsp90) by natural products: geldanamycin, a show case in cancer therapy. *Nat Prod Rep.* **30**, 1299-1323; 10.1039/c3np70012g (2013).

Cx26 protein synthesis.

Cx26K188N was generated according to a DpnI based protocol described recently^56^.

DNA sequence of *E. coli* codon usage adapted Cx26WT:

ATGGATTGGGGTACTCTGCAGACGATTCTGGGTGGAGTCAACAAACATTCGACAAGCATTGGGAAAATTTGGTTGACCGTTCTGTTTATCTTCCGGATTATGATTCTCGTTGTAGCCGCGAAAGAAGTTTGGGGTGATGAACAGGCAGACTTCGTCTGCAATACCTTGCAACCGGGGTGTAAGAACGTCTGTTATGACCACTACTTTCCGATCTCACACATTCGTCTTTGGGCGTTACAGCTGATCTTTGTCTCCACACCCGCTTTACTGGTTGCCATGCATGTGGCGTATCGTCGCCATGAAAAGAAACGCAAGTTTATCAAAGGCGAAATCAAATCCGAGTTTAAAGACATTGAAGAGATCAAAACGCAGAAAGTACGCATTGAAGGCTCTCTGTGGTGGACTTACACGAGTAGCATCTTCTTTCGTGTGATTTTCGAAGCTGCGTTTATGTATGTCTTTTACGTTATGTATGATGGCTTCAGCATGCAACGCCTGGTGAAATGCAATGCATGGCCTTGTCCGAATACGGTGGATTGCTTCGTGTCTCGTCCAACCGAGAAAACCGTGTTTACCGTGTTCATGATTGCCGTATCGGGCATTTGCATCCTGCTTAACGTTACCGAACTGTGCTATCTGCTCATTCGCTACTGTAGTGGTAAAAGCAAGAAACCGGTGTAA

Primer generating the Cx26K188N mutant.

Cx26K188N for: TCC AAC CGA GAA TAC CGT GTT TAC (T_M_=55.7 °C)

Cx26K188N rev: GTA AAC ACG GTA TTC TCG GTT GGA (T_M_=55.7 °C)
